# Polynucleotide HPT^TM^-Based Hydrogels Exhibit Scavenging Activity Against Reactive Oxygen Species

**DOI:** 10.3390/antiox14091089

**Published:** 2025-09-05

**Authors:** Maria Teresa Colangelo, Silvana Belletti, Stefano Guizzardi, Carlo Galli

**Affiliations:** Histology and Embryology Laboratory, Department of Medicine and Surgery, University of Parma, Via Volturno 39, 43126 Parma, Italy; mariateresa.colangelo@unipr.it (M.T.C.); silvana.belletti@unipr.it (S.B.); stefano.guizzardi@unipr.it (S.G.)

**Keywords:** polynucleotides, hyaluronic acid, oxidative stress, scavenger activity, antioxidant therapy

## Abstract

This study investigates the scavenger activity of Polynucleotide High Purification Technology (PN HPT^TM^), alone or in combination with hyaluronic acid (PN HPT^TM^ + HA) against oxidative stress induced by hydrogen peroxide (H_2_O_2_). Since oxidative stress is implicated in numerous pathological conditions, identifying effective antioxidants is crucial for therapeutic development. We employed a cell-free fluorometric assay based on Calcein-AM, a fluorescence probe whose signal increases proportionally to the generation of reactive oxygen species (ROS), to evaluate the ability to neutralize ROS under varying oxidative stress conditions and determine the dose- and time-dependent effects of these compounds. PN HPT^TM^, HA, and PN HPT^TM^ + HA were tested at various concentrations over multiple time points. Our results demonstrated that all tested treatments significantly lowered ROS levels compared to the untreated control. Notably, the PN HPT^TM^ -based compounds exhibited robust scavenging activity, with PN HPT^TM^ + HA displaying the strongest and most consistent ROS-neutralizing effect across all concentrations and time points. This enhanced performance suggests a synergistic interaction between PN HPT^TM^ and HA, potentially due to complementary mechanisms of free radical scavenging and structural stabilization. These findings highlight the potential of PN HPT^TM^ and PN HPT^TM^ + HA as effective antioxidative agents, offering potential for therapeutic applications where oxidative stress is central, including wound healing and tissue regeneration.

## 1. Introduction

Reactive oxygen species (ROS) are a diverse family of oxidants derived from molecular oxygen during cell processes such as respiration and include both radical species like superoxide (O_2_^−^) and hydroxyl radicals ( OH), as well as non-radical molecules such as hydrogen peroxide (H_2_O_2_) [1]. Under normal physiological conditions, ROS are necessary components to various cellular processes, such as signaling and metabolic pathways, and the maintenance of redox homeostasis [1,2]. These physiological levels of ROS contribute to what is known as oxidative eustress, which is essential for regular cell function by activating transcription factors like nuclear factor-κB (NF-κB) and mitogen-activated protein kinase (MAPK) cascades [3]. However, if the production of ROS exceeds the capacity of cellular antioxidant defenses, the resulting imbalance may lead to oxidative stress which causes cell and tissue damage [4].

One significant target of ROS during oxidative stress is the extracellular matrix (ECM). The ECM is a complex network of structural proteins such as collagens and elastin, glycoproteins like fibronectin, and proteoglycans, including glycosaminoglycans like hyaluronic acid (HA) [5]. Beyond providing structural support to tissues, the ECM regulates crucial processes, including cell adhesion, migration, proliferation, and differentiation [6,7]. Chemical modifications to ECM components caused by ROS can severely impact tissue integrity and repair [8,9]. For example, oxidative damage to collagen fibers compromises their structural stability, making them more susceptible to enzymatic degradation [10]. Similarly, alterations to HA can result in increased fragmentation and in the loss of its viscoelastic and hydrating properties [11]. These changes disrupt the ECM’s ability to mediate cell signaling and bind growth factors, further impairing tissue regeneration and repair [12].

ECM degradation is particularly pronounced in chronic inflammatory conditions such as periodontitis, aging, and impaired wound healing [13]. Excessive ROS production destabilizes the delicate balance between ECM synthesis and turnover, initiating destructive processes [14]. For instance, in the gingival tissues affected by periodontitis, ROS-induced modifications to proteoglycans alter their core proteins and glycosaminoglycan chains, impairing their ability to regulate tissue homeostasis [15].

Addressing ROS-induced ECM damage is crucial, given the ECM’s pivotal role in maintaining tissue function and facilitating repair. Numerous research efforts are actively focused on this challenge [16,17,18,19]. However, effective solutions must go beyond neutralizing ROS; they must also be designed to provide biochemical signals and structural support that favor tissue regeneration. To this effect, interventions capable of reestablishing a regenerative microenvironment under oxidative stress conditions are needed [20,21,22].

Biomaterial scaffolds are particularly well-suited for this purpose, as they are a commonly used strategy to promote tissue repair and regeneration [23,24]. In particular, hydrogels have attracted attention as promising tools in tissue engineering [25,26,27,28]. Hydrogels are versatile, three-dimensional networks capable of mimicking the natural structure and function of the ECM [29]. They are highly biocompatible and possess unique physicochemical properties, including high water content and porosity, making them ideal scaffolds for supporting cell adhesion and viability [30,31,32,33,34]. By providing a biomimetic microenvironment, hydrogels facilitate cellular processes that are essential for tissue repair and regeneration [35].

Recent advances have produced antioxidant hydrogels that actively buffer ROS while supporting repair, including ECM-mimetic or cardiac matrix hydrogels with intrinsic scavenging capacity and self-assembling glycopeptide systems that quench ROS and modulate inflammation [18,19,36]. In parallel, HA functionalization strategies—thiolation, catechol/phenylboronate grafting, and ROS-responsive linkers—are being used to endow HA networks with adhesive or ROS-cleavable behavior, and nucleic-acid–based scaffolds (e.g., framework nucleic acids) are emerging as direct ROS scavengers and delivery platforms [37]. Considering these recent advances, we specifically probe, under cell-free conditions, the intrinsic radical-scavenging capacity of Polynucleotides and Hyaluronic acid (HA), alone and in combination.

Polynucleotides (PN) HPT^TM^ and HA have shown significant potential as components of hydrogel scaffolds due to their combined biochemical and structural contributions. PN HPT^TM^, derived from DNA fragments, support cell vitality by providing a stable and physiological microenvironment [38,39]. HA, a major glycosaminoglycan in the ECM, is known for its viscoelasticity, hydrating properties, and useful scaffold characteristics to modulate tissue repair [36,40,41,42]. More recently, products with PN HPT^TM^ and with PN HPT^TM^ combined with HA (PN HPT^TM^ + HA) have been proposed for improved tissue conditions and have demonstrated considerable clinical potential [43,44,45,46,47,48,49,50,51,52,53].

Despite these advancements, the potential of PN HPT^TM^ and PN HPT^TM^ + HA hydrogels for mitigating oxidative stress has not been explored. We hypothesize that these hydrogels can effectively reduce ROS levels and thereby help preserve ECM integrity under oxidative stress conditions. Therefore, in this brief report, we utilized a cell-free in vitro model of H_2_O_2_-induced oxidative stress to assess the direct ROS-scavenging capabilities of these hydrogels. This work provides a standardized, solvent-free, cell-free, kinetic benchmark of clinical-grade PN HPT™ and HA—alone or in combination—across oxidant dose and time, anchored to an in-assay N-acetyl-L-cysteine (NAC) titration and complemented by ORAC antioxidant assay, establishing a robust baseline for subsequent cellular and structural studies.

## 2. Materials and Methods

### 2.1. Polynucleotide High Purification Technology (PN HPT^TM^)

Polynucleotide High Purification Technology (PN HPT^TM^) used in this study were obtained from Mastelli S.r.l. (Sanremo, Italy). PN HPT^TM^ is a compound containing DNA fragments of varying chain lengths, extracted from the gonads of salmon trout (*Oncorhynchus mykiss*) through an original high-purification technology (HPT™). This technology provides high-quality DNA while minimizing immunological side effects [34]. The products employed in the present study are commercially available Class III medical device hydrogels: PN HPT^TM^ (20 mg/mL), hyaluronic acid (HA) (20 mg/mL), and a combination of PN HPT^TM^ and HA (PN HPT^TM^ + HA) containing 10 mg/mL PN HPT^TM^ and 10 mg/mL HA.

### 2.2. H_2_O_2_ Oxidative Stress Assay

The scavenger activity of PN HPT™, HA, and PN HPT™ + HA against H_2_O_2_-driven ROS was evaluated in two experimental settings using a cell-free fluorometric assay with Calcein-AM fluorescence as a marker for oxidative stress.

In the first set of experiments, the antioxidant capacity of each compound at the maximal commercially available concentration (undiluted, indicated as “Max” in the figures) was assessed against increasing concentrations of hydrogen peroxide (0, 150, 300, 600, and 1000 μM) to determine their ability to neutralize reactive oxygen species (ROS) under varying oxidative stress conditions. Aliquots of 100 μL of each compound were dispensed into a 96-well black microplate (Thermo-Fisher); phosphate-buffered saline (PBS, Sigma-Aldrich, St. Louis, MO, USA) serving as the control (indicated as 0 μg/mL). Calcein-AM (2 μM; Thermo-Fisher, Waltham, MA, USA) was added to each well, followed by a 10 min incubation at room temperature to allow fluorescence development. H_2_O_2_ was then introduced to induce oxidative stress, and fluorescence was recorded after 60 min at an emission wavelength of 530 nm using a microplate reader (Infinite F200, Tecan, Männedorf, Switzerland). Data were acquired with Tecan i-Control software, version 1.5.

In the second set of experiments, the same assay protocol was applied to test the compounds at multiple concentrations (Max, 900, 600, and 300 μg/mL in PBS) against a fixed H_2_O_2_ concentration (600 μM). N-acetyl-L-cysteine (NAC; Sigma-Aldrich, St. Louis, MO, USA) was used as a positive control at 1.25, 2.5, and 5 mM concentrations to provide a quantitative benchmark for scavenging activity. This setup enabled the evaluation of dose- and time-dependent effects, with fluorescence measurements taken at 10, 30, 60, 90, and 120 min.

### 2.3. ORAC Assay

The oxygen-radical absorbance capacity (ORAC) was measured as described by Huang et al. [54], in 75 mM PBS (pH 7.4) at 37 °C using an Infinite F200 (Tecan, Switzerland) with I-Control software. In black 96-well plates, 150 µL fluorescein (80 nM final) and 25 µL of samples were pre-incubated for 30 min, then peroxyl-radical generation was initiated with 25 µL of 2,2-Azobis(2-methylpropionamidine) dihydrochloride (AAPH; Sigma-Aldrich, St. Louis, MO, USA; 153 mM). Fluorescence (Ex 485 nm/Em 535 nm) was recorded every minute for 30 min. All measurements were performed in 5 replicate wells per treatment with light protection.

### 2.4. Statistical Analysis

Calcein fluorescence data were reported as mean ± standard deviation. Differences between groups in the dose–response experiments were assessed using two-way ANOVA followed by Bonferroni’s post hoc test to compare treated samples against the control (Prism v10.2.3, GraphPad, La Jolla, CA, USA).

For the ORAC assay, the area under the fluorescence–time curve (AUC) was calculated for each replicate using the trapezoidal method. Group differences in AUC were analyzed by one-way ANOVA, with *p*-values adjusted for multiple comparisons using Tukey’s post hoc test. Additionally, fluorescence values were compared at each minute across treatments using one-way ANOVA with Tukey’s post hoc test to identify the earliest time point at which PN HPT™ + HA became significantly higher than PN HPT™ or HA alone.

Statistical significance was set at *p* < 0.05 and each experiment was repeated three times to ensure reproducibility.

## 3. Results and Discussion

The purpose of our investigation was to assess whether Polynucleotide High Purification Technology (PN HPT^TM^) with or without Hyaluronic Acid (HA) could hamper ROS under challenging conditions that mimic the oxidative stress associated with harmful tissue and organ conditions [55]. For this purpose, we relied on a model we previously developed and chacterized to evaluate H_2_O_2_-induced ROS [56]. This approach allowed us to focus on direct ROS-scavenging activity, avoiding confounding variables such as cellular metabolism or signaling.

**Figure 1:**
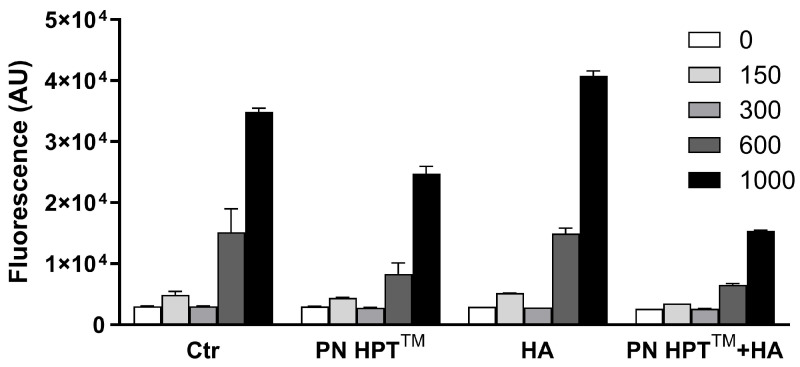
Dose–response relationship between hydrogen peroxide (H_2_O_2_)-derived reactive-oxygen-species (ROS) and their modulation by Polynucleotide High Purification Technology (PN HPT™), hyaluronic acid (HA), and the combined formulation (PN HPT™ + HA). Increasing H_2_O_2_ concentrations—0, 150, 300, 600, and 1000 µM—were added to the samples for 60 min, either in the absence (Ctr) or in the presence of each compound supplied at its highest test concentration. ROS accumulation was quantified as fluorescence and expressed in arbitrary units (AU); bars represent mean ± SD of three independent experiments. See Appendix A for statistical analysis.

To define a working oxidative challenge, we first exposed the compounds to an escalating dose of H_2_O_2_ (0–1000 µM) and quantified ROS--dependent fluorescence in the absence (Ctr) or presence of the test materials using the Calcein-AM probe (Figure 1).

In control samples, fluorescence rose almost linearly with the oxidant load, climbing from basal values at 0 µM to about 2 × 10^4^ AU at 600 µM and peaking near 4 × 10^4^ AU at 1000 µM (*p* < 0.0001, two-way ANOVA). All three formulations attenuated this response, but with different efficacies: PN HPT™ and HA alone produced intermediate, dose-dependent reductions, whereas their combination (PN HPT™ + HA) provided the strongest protection, keeping the signal around 1 × 10^4^ AU even at the highest peroxide dose. See more details about the statistical analysis of these data in Appendix A.

We selected 600 µM H_2_O_2_ for all following experiments because it elicited a robust yet sub-maximal signal that clearly discriminated among treatments without saturating the read-out.

**Figure 2:**
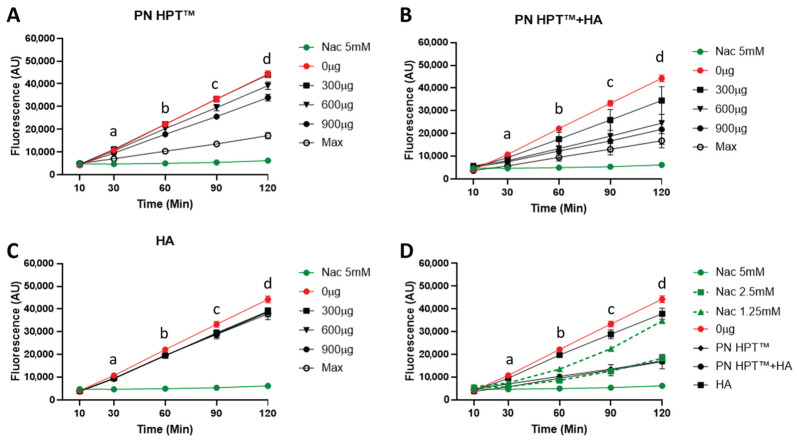
Scavenger activity of Polynucleotide High Purification Technology (PN HPT™), hyaluronic acid (HA), and their combination (PN HPT™ + HA) against H_2_O_2_-induced ROS, monitored as calcein fluorescence at 10, 30, 60, 90, and 120 min. Panel (**A**) shows PN HPT™ at 0, 300, 600, and 900 μg mL^−1^ and at its maximal commercial concentration (“Max”); panels (**B**,**C**) present PN HPT™ + HA and HA, respectively, under the same concentration regimen; in each case 5 mM N-acetyl-cysteine (NAC; green) serves as the positive antioxidant control. Panel (**D**) compares the three materials at their Max concentrations with a NAC titration (1.25, 2.5, and 5 mM) and the oxidant control (0 μg mL^−1^). Across doses and time points, both PN HPT™ and PN HPT™ + HA reduced the H_2_O_2_-driven fluorescence versus the oxidant control; the mixture tended to yield lower signals than PN HPT™ alone at matched doses, although at the highest concentration the two curves overlapped within the SD. Data are mean ± SD (n = 3). See Appendix A for the statistical analysis.

Figure 2 illustrates fluorescence levels measured using the Calcein-AM probe for PN HPT^TM^ (Figure 2A), PN HPT^TM^ + HA (Figure 2B), and HA (Figure 2C) at different concentrations of these compounds (300, 600, and 900 μg/mL, including the maximum concentration) over time (10, 30, 60, 90, and 120 min after H_2_O_2_ addition). NAC at 5 mM concentration (green line) provided a positive control of a known antioxidant.

As visible in Figure 2, the rising intensity of Calcein-AM fluorescence in the control group (0 μg/mL, red line) indicated that ROS levels progressively increased over time after addition of H_2_O_2_. PN HPT^TM^ exhibited a strong, dose-dependent scavenging activity, as evidenced by significantly reduced fluorescence at concentrations ≥ 900 μg/mL (Figure 2A). At the highest concentration, PN HPT^TM^ significantly lowered fluorescence as early as 30 min after H_2_O_2_ exposure. Lower concentrations produced statistically significant reductions later, around 60–120 min. Throughout the 120 min recordings, samples remained fully soluble and optically clear under the neutral, aqueous conditions used, minimizing the likelihood of measurement artefacts related to gelation or light scattering. These observations indicate the robust antioxidant properties of PN HPT^TM^ compounds against H_2_O_2_-derived ROS and suggest that sufficient PN HPT^TM^ concentration can rapidly and effectively neutralize free radicals. This aligns with reports suggesting that nucleotides or nucleotide-containing fragments can act as scavengers for multiple ROS, possibly by directly donating electrons or forming stable complexes that inhibit radical propagation [37].

The PN HPT^TM^ + HA combination demonstrated the most pronounced reduction in fluorescence intensity across all time points (Figure 2B). At 60–120 min, PN HPT^TM^ + HA significantly reduced ROS levels compared to the control, even at 600 μg/mL. At matched doses, PN HPT™ + HA generally produced lower fluorescence than PN HPT™ alone, but at the Max concentration the trajectories largely overlapped and pairwise differences were not significant (Appendix A).

Hyaluronic acid (HA) alone also reduced fluorescence intensity compared to the control group, though its effects were less pronounced than those of PN HPT^TM^ or PN HPT^TM^ + HA (Figure 2B). HA’s scavenging activity was not strongly concentration-dependent, with statistically significant reductions observed starting at 90 min and only for the concentration of 600 μg/mL. Albeit limitedly, HA reduced ROS levels; however, its scavenging activity remained lower than that of PN HPT^TM^ products at any concentration.

The enhanced activity of PN HPT™ + HA may result from complementary mechanisms of PN HPT^TM^ and HA; we hypothesize that while specific chemical groups in PN HPT^TM^ (e.g., nitrogenous bases) can directly interact with and neutralize free radicals, HA’s large, charged glycosaminoglycan structure might provide multiple additional reactive sites for binding or quenching ROS. However, the peculiar behavior of PN HPT^TM^ + HA could also be centered on HA’s high water-binding capacity, which could create a hydrophilic microenvironment that dilutes ROS or slows their diffusion, thus reducing their local concentration. Collectively, these properties suggest a synergistic interaction in which HA’s structural and hydrating functions support and amplify PN HPT^TM^’s direct radical-scavenging potential. At physiological pH, both polymers behave as polyanions surrounded by extensive hydration shells and mobile counter-ions, which increase local microviscosity and can slow diffusion-limited radical reactions, providing a physicochemical basis for a water-rich microenvironment. In addition, purine bases—especially guanine—have the lowest oxidation potential among nucleobases and undergo one-electron oxidation to delocalized radical cations that evolve to 8-oxo-guanine [57], supplying a sacrificial electron-donation pathway consistent with the strong performance of PN HPT™ against H_2_O_2_-driven ROS. Under the mild, short-timescale oxidative challenges applied here, both HA and PN HPT™ are expected primarily to undergo sacrificial reactions—HA by hydrogen abstraction, ring opening and chain scission [58], and PN HPT™ by nucleobase oxidation (guanine) and occasional backbone breaks—rather than spontaneous inter-polymer crosslinking [57] (Figure S1). The combination’s efficacy can therefore be explained by complementary radical chemistry and hydration-mediated diffusion effects.

In the direct head-to-head comparison (Figure 2D), the fluorescence trajectories elicited by PN HPT™ and by the PN HPT™ + HA mixture overlapped almost perfectly with the curve generated by 2.5 mM NAC, remaining well below the signals obtained with 1.25 mM NAC and only marginally higher than the 5 mM NAC reference throughout the 120 min recording. Two-way ANOVA confirmed that at every time point, PN HPT™ and PN HPT™ + HA did not differ significantly from 2.5 mM NAC (*p* > 0.05), while both were significantly more effective than HA alone and the oxidant control (*p* < 0.01). These data indicate that, at their maximal test concentrations, PN HPT™ and its combination with HA confer an antioxidant protection quantitatively equivalent to an intermediate NAC dose of 2.5 mM, underscoring their capacity to curb peroxide-driven ROS generation to a level comparable with a well-established thiol antioxidant.

Although direct precedents for a cell-free Calcein-AM ROS read-out are scarce, our kinetics and pharmacologic controls (NAC titration) align with reports that use calcein/dihydrocalcein probes in cells, where oxidative stress drives a monotonic fluorescence increase that is blunted by reducing/antioxidant conditions [56], thus corroborating our Calcein-AM data. Buskiewicz et al. tracked ROS with dihydrocalcein in mammalian cells and showed that antioxidants suppress the signal in parallel with redox-sensitive mitochondrial changes, reinforcing the specificity of the read-out for oxidative activity [59]. Likewise, Rohnstock et al. used dihydrocalcein oxidation to calcein to quantify intracellular oxidative bursts, again demonstrating inhibitor sensitivity consistent with a ROS-dependent mechanism [60].

**Figure 3:**
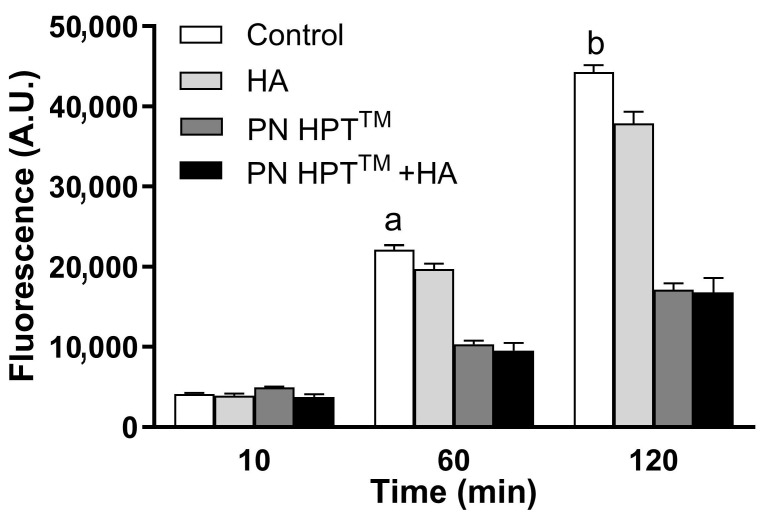
Scavenger activity of Control, HA, PN HPT^TM^, and PN HPT^TM^ + HA products in response to H_2_O_2_-induced oxidative stress. Fluorescence intensity (A.U.) was measured at 10, 60, and 120 min for the vehicle (control), PN HPT^TM^, HA, and PN HPT^TM^ + HA at maximum concentration. PN HPT^TM^ and PN HPT^TM^ + HA significantly reduced fluorescence compared to the vehicle at 60 min (*p* < 0.001, “a”), and at 120 min (*p* < 0.001, “b”). Data are expressed as mean ± SD.

Figure 3 illustrates fluorescence levels at the highest compound concentrations at 10, 60, and 120 min post-H_2_O_2_ exposure, highlighting the superior scavenging efficacy of PN HPT^TM^ and PN HPT^TM^ + HA over HA alone.

**Figure A1:**
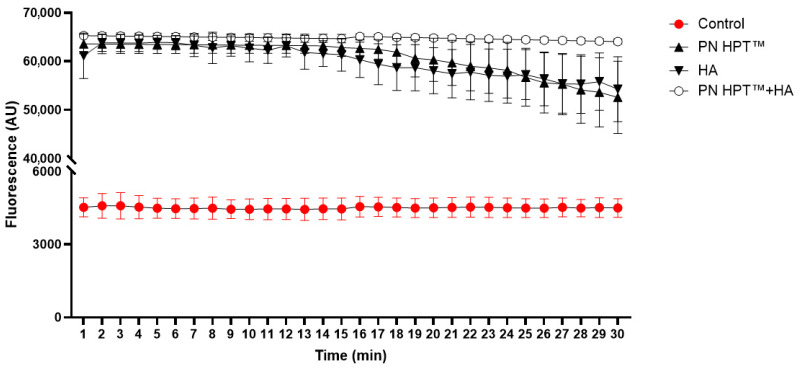
Oxygen-radical absorbance capacity (ORAC) kinetics of Polynucleotide High Purification Technology (PN HPT™), hyaluronic acid (HA) and their combination (PN HPT™ + HA) compared with the radical control. In a cell-free phosphate buffer (75 mM, pH 7.4, 37 °C) fluorescein (80 nM) was challenged with peroxyl radicals generated by AAPH (153 mM) and fluorescence was recorded at 485/535 nm every minute for 30 min. Antioxidants were supplied at the maximal test concentration used throughout the study immediately before radical generation. Curves represent the mean ± SD of three independent experiments; higher fluorescence indicates more effective scavenging of peroxyl radicals. The PN HPT™ + HA group became statistically higher than both the HA and PN HPT™ groups starting at minute 16.

ORAC, a complementary antioxidant assay, carried out in analogous cell-free conditions as those adopted for the Calcein assay, substantiated the overall antioxidant potential of the tested compounds, yet disclosed a distinct hierarchy (Figure A1). Analysis of the fluorescence curves by area under the curve (AUC) confirmed significant differences among compounds (one-way ANOVA, F(3,16) = 749.03, *p* = 2.08 × 10^−17^). Tukey’s post hoc test showed that PN HPT™ + HA had the highest scavenging capacity, significantly exceeding both PN HPT™ and HA alone (*p* < 0.0001 for both comparisons). Time-resolved analysis indicated that the fluorescence in the PN HPT™ + HA group became significantly higher than in the other two treatments starting from minute 16 on, sustaining this difference until the end of the assay (Figure A1 and Table A1).

Unlike the Calcein assay, however, no difference was detected between PN HPT™ alone and HA. This divergence from the Calcein read-out can be ascribed to the different radical species generated in the two tests. In the Calcein system, Guanine-rich PN HPT™ can be hypothesized to be particularly effective because guanine has the lowest redox potential among the nucleobases and is readily oxidised to 8-oxo-guanine, sacrificially intercepting hydroxyl radicals generated by H_2_O_2_. In contrast, the ORAC protocol relies on thermolysis of AAPH to yield peroxyl radicals that propagate by hydrogen abstraction along organic chains; such chain-breaking events may be more effectively inhibited by HA’s numerous saccharide hydroxyls and by the steric shielding conferred when HA and PN HPT™ are combined, explaining the superior performance of the PN HPT™ + HA combination and the relative levelling of PN HPT™ and HA alone.

The implications of these findings are significant, given the central role of oxidative stress in the pathogenesis of numerous conditions, including aging [4,61,62,63], periodontitis [64,65], and osteoarthrosis [66,67,68,69,70]. This study demonstrates antioxidant activity of PN HPT^TM^ and PN HPT^TM^ + HA products, positioning them as promising candidates for therapeutic applications aimed at locally reducing oxidative damage. Their robust activity against ROS could also partially explain the favourable clinical outcomes observed with PN HPT^TM^ -containing products across diverse clinical contexts, including dermatology, wound healing, gynecology, osteoarthrosis, dentistry and aesthetic medicine [46,47,48,49,50,51,52,53,71,72,73,74].

Thus, our head-to-head profiling across oxidant classes identifies complementary radical selectivity: PN HPT™ performs best under H_2_O_2_-driven conditions consistent with ·OH interception by nucleobases, while HA may favor peroxyl-radical quenching, and PN HPT™ + HA performs best overall. This comparative insight has not been reported previously for clinical-grade PN/HA materials and offers a mechanistic rationale for outcomes described in clinical studies using PN, HA, or their combination where mixed ROS milieus are expected. Moreover, anchoring the effect size to a NAC titration (≈2.5 mM equivalence) provides a usable quantitative yardstick for future materials.

While advanced platforms (e.g., ROS-responsive HA networks, self-assembling glycopeptides, or framework-nucleic-acid scaffolds) introduce designed triggers or catalytic motifs [18,19,37,59,60], the present data establish a clean materials baseline for PN-based and HA-based formulations and justify deeper, mechanism-aware studies that integrate these chemistries.

Nevertheless, our approach has limitations. While the cell-free system effectively isolates scavenging activity, it does not recapitulate the complexity of living tissues, where factors like enzymatic activity, local pH, and cellular signaling may influence ROS levels. Further in vitro and in vivo experiments are needed to clarify how PN HPT^TM^ and PN HPT^TM^ + HA functions within the physiological milieu, how quickly it is metabolized or replaced, and whether its protective effects extend to diverse cell types and tissues. We note that prior calcein/dihydrocalcein literature is predominantly cellular, with very limited cell-free implementations; our data therefore establish a clean baseline for Calcein-AM–based ROS scavenging in a strictly cell-free system and complement those intracellular precedents.

Investigating the molecular details of how PN HPT™ and HA interact with each other, as well as whether they engage with endogenous antioxidant systems, will be an important focus for future studies.

## 4. Conclusions

This study demonstrates significant antioxidant properties of Polynucleotide High Purification Technology (PN HPT™), especially in combination with HA (PN HPT™ + HA), in cell-free models of oxidative stress. Both Calcein-AM and ORAC assays consistently showed that PN HPT™ + HA reduce ROS activity in a dose- and time-dependent manner. The synergistic activity observed in the PN HPT™ + HA combination underscores its potential as a powerful therapeutic tool for mitigating oxidative damage. These results suggest promising applications in medical and aesthetic fields, particularly in contexts such as tissue repair, wound healing, osteoarthrosis, and anti-aging therapies. Further research is needed to validate these findings in more complex biological systems and to elucidate the mechanisms underlying their antioxidant effects. The insights provided by this study form a solid foundation for advancing the development of PN HPT™ and PN HPT™ + HA as innovative solutions to oxidative stress-related challenges.

## Data Availability

The data presented in this study are available on request from the corresponding author.

## Supplementary Materials

The following supporting information can be downloaded at: https://www.mdpi.com/article/10.3390/antiox14091089/s1, Figure S1: Chemical structures of (A) Polynucleotides, showing representative DNA fragments composed of multiple nucleotides linked by 3′,5′-phosphodiester bonds, with the four nitrogenous bases indicated (adenine, guanine, cytosine, and thymine), (B) Hyaluronic acid, a linear polysaccharide consisting of repeating disaccharide units of D-glucuronic acid and N-acetyl-D-glucosamine linked by β(1→3) and β(1→4) glycosidic bonds.

## Appendix A

Scavenging activity of Polynucleotide High Purification Technology (PN HPT^TM^), Hyaluronic acid (HA), or Polynucleotides HPT^TM^ + Hyaluronic Acid (PN HPT^TM^ + HA) compounds in response to H_2_O_2_-induced oxidative stress.

Results of Tukey’s post-test for the experiment in Figure 1 are presented below.

600 μM H_2_O_2_

a: *p* < 0.0001 Control vs. PN HPT™ and Control vs. PN HPT™ + HA

c: *p* < 0.0001 PN HPT™ vs. HA

e: *p* < 0.0001 HA vs. PN HPT™ + HA

1000 μM H_2_O_2_

b: *p* < 0.0001 Control vs. PN HPT™, HA, and PN HPT™ + HA.

d: *p* < 0.0001 PN HPT™ vs. HA, and PN HPT™ + HA

f: *p* < 0.0001 HA vs. PN HPT™ + HA

Results of the Bonferroni post-test for the experiments in Figure 2 are:

PN HPT^TM^:

30′: a *p* = 0.002 0 μg/mL vs. Max concentration; *p* = 0.003 NAC vs. 0, 300, 600 μg/mL, *p* = 0.05 NAC vs. 900 μg/mL.

60′: b *p* = 0.0004 0 μg/mL vs. Max concentration, *p* = 0.01 0 μg/mL vs. 900 μg/mL, *p* = 0.001 NAC vs. 0, 300, 600, 900 μg/mL, *p* = 0.01 NAC vs. Max concentration.

90′: c *p* = 0.0002 0 μg/mL vs. Max concentration, *p* = 0.005 0 μg/mL vs. 900 μg/mL, *p* = 0.001 NAC vs. 0, 300, 600, 900 μg/mL, *p* = 0.01 NAC vs. Max concentration.

120′: d *p* < 0.001 0 μg/mL vs. Max concentration, *p* = 0.004 0 μg/mL vs. 900 μg/mL, *p* = 0.001 NAC vs. 0, 300, *p* = 0.002 NAC vs. 600, 900 μg/mL, *p* = 0.02 NAC vs. Max concentration.

HA

30′: a *p* = 0.001 NAC vs. 0, 300, 600, 900 μg/mL, *p* = 0.02 NAC vs. Max concentration

60′: b *p* = 0.001 NAC vs. 0, 300, 600, 900 μg/mL, *p* = 0.01 NAC vs. Max concentration

90′: c *p* = 0.05 0 μg/mL vs. 600 μg/mL.

120′: d *p* = 0.04 0 μg/mL vs. 600 μg/mL.

PN HPT^TM^ + HA

30′: a *p* = 0.01 0 μg/mL vs. Max concentration, *p* = 0.02 0 μg/mL vs. 900 μg/mL, *p* = 0.001 NAC vs. 0 μg/mL.

60′: b *p* = 0.004 0 μg/mL vs. Max concentration, *p* = 0.006 0 μg/mL vs. 900 μg/mL, *p* = 0.02 0 μg/mL vs. 600 μg/mL, *p* = 0.001 NAC vs. 0 μg/mL, *p* = 0.05 vs. 300 μg/mL.

90′: c *p* = 0.004 0 μg/mL vs. Max concentration, *p* = 0.002 0 μg/mL vs. 900 μg/mL and 0 μg/mL vs. 600 μg/mL, *p* = 0.001 NAC vs. 0 μg/mL, *p* = 0.05 vs. 300 μg/mL.

120′: d *p* = 0.004 0 μg/mL vs. Max concentration, *p* = 0.001 0 μg/mL vs. 900 μg/mL, *p* = 0.01 0 μg/mL vs. 600 μg/mL, *p* = 0.001 NAC vs. 0 μg/mL, *p* = 0.05 vs. 300 μg/mL.

NAC vs. Max concentration compounds

30′: a *p* = 0.005 NAC 5 mM vs. 0 μg/mL, *p* = 0.01 NAC 5 mM vs. HA, *p* = 0.03 NAC 2.5 mM vs. 0 μg/mL.

60′: b *p* = 0.001 NAC 5 mM vs. 0 μg/mL, *p* = 0.01 NAC 5 mM vs. HA, *p* = 0.01 NAC 2.5 mM vs. 0 μg/mL.

90′: c *p* = 0.001 NAC 5 mM vs. 0 μg/mL, *p* = 0.03 NAC 5 mM vs. HA, *p* = 0.003 NAC 2.5 mM vs. 0 μg/mL, *p* = 0.03 NAC 1.25 mM vs. 0 μg/mL.

120′: d *p* = 0.001 NAC 5 mM vs. 0 μg/mL, *p* = 0.03 NAC 5 mM vs. HA, *p* = 0.002 NAC 2.5 mM vs. 0 μg/mL, *p* = 0.05 NAC 1.25 mM vs. 0 μg/mL.

## Appendix B

**Table A1:** Area under the fluorescence–time curve (AUC, arbitrary units) for each treatment group in the ORAC assay. Data are mean ± SD of five replicates per group. One-way ANOVA revealed a significant effect of treatment (F(3,16) = 749.03, *p* = 2.08 × 10^−17^).

Treatment	AUC
Control	130,609 ± 12,069
HA	1,743,405 ± 97,672
PN HPT™	1,763,275 ± 93,434
PN HPT™ + HA	1,878,686 ± 13,199
