# Supplementary material for: Polynucleotide HPTTM-Based Hydrogels Exhibit Scavenging Activity Against Reactive Oxygen Species
Source: Antioxidants (Basel). 2025 Sep 5;14(9):1089. doi: 10.3390/antiox14091089 (PMC12466773; doi:10.3390/antiox14091089)
Supplement: Supplementary file 1 [file antioxidants-14-01089-s001.zip › antioxidants-3695961-supplementary.pdf]

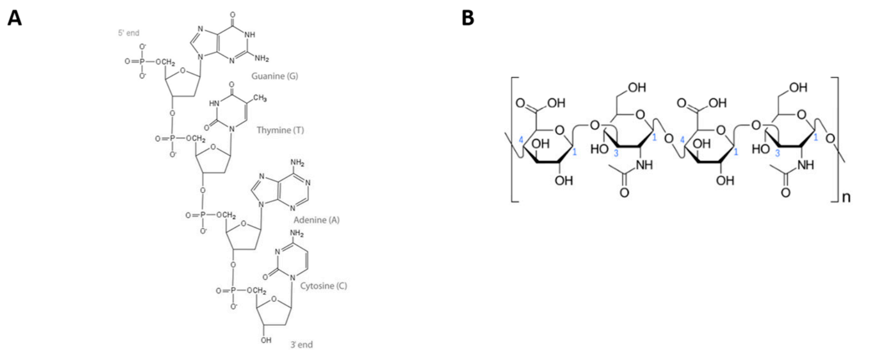

Figure S1. Chemical structures of (A) Polynucleotides, showing representative DNA fragments composed of multiple nucleotides linked by 3′,5′-phosphodiester bonds, with the four nitrogenous bases indicated (adenine, guanine, cytosine, and thymine), (B) Hyaluronic acid, a linear polysaccharide consisting of repeating disaccharide units of D-glucuronic acid and N-acetyl-D-glucosamine linked by β(1→3) and β(1→4) glycosidic bonds.
